# The effect of mechanical loads on the degradation of aliphatic biodegradable polyesters

**DOI:** 10.1093/rb/rbx009

**Published:** 2017-04-17

**Authors:** Ying Li, Zhaowei Chu, Xiaoming Li, Xili Ding, Meng Guo, Haoran Zhao, Jie Yao, Lizhen Wang, Qiang Cai, Yubo Fan

**Affiliations:** 1 School of Biological Science and Medical Engineering, Key Laboratory for Biomechanics and Mechanobiology of Ministry of Education, International Research Center for Implantable and Interventional Medical Devices, Beihang University, Beijing 100191, People’s Republic of China;; 2 Department of Biomedical Engineer, University of Cincinnati, Cincinnati, OH 45221, USA;; 3 Key Laboratory of Advanced Materials of Ministry of Education of China, Tsinghua University, Beijing 100084, People’s Republic of China;; 4 National Research Center for Rehabilitation Technical Aids, Beijing 100176, People’s Republic of China

**Keywords:** aliphatic biodegradable polyesters, mechanical load, degradation

## Abstract

Aliphatic biodegradable polyesters have been the most widely used synthetic polymers for developing biodegradable devices as alternatives for the currently used permanent medical devices. The performances during biodegradation process play crucial roles for final realization of their functions. Because physiological and biochemical environment *in vivo* significantly affects biodegradation process, large numbers of studies on effects of mechanical loads on the degradation of aliphatic biodegradable polyesters have been launched during last decades. In this review article, we discussed the mechanism of biodegradation and several different mechanical loads that have been reported to affect the biodegradation process. Other physiological and biochemical factors related to mechanical loads were also discussed. The mechanical load could change the conformational strain energy and morphology to weaken the stability of the polymer. Besides, the load and pattern could accelerate the loss of intrinsic mechanical properties of polymers. This indicated that investigations into effects of mechanical loads on the degradation should be indispensable. More combination condition of mechanical loads and multiple factors should be considered in order to keep the degradation rate controllable and evaluate the degradation process *in vivo* accurately. Only then can the degradable devise achieve the desired effects and further expand the special applications of aliphatic biodegradable polyesters.

## Introduction

**Figure 1.**
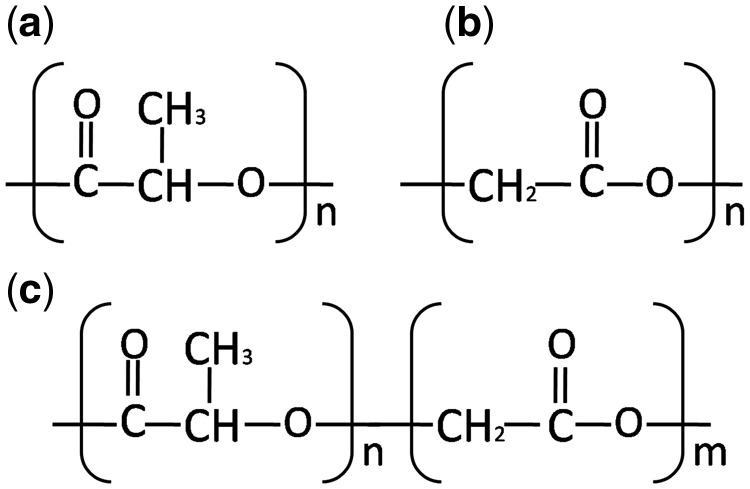
Structure of **(a)** PLA, **(b)** PGA and **(c)** PLGA

With the development of degradable biomaterials science during the last decades, biodegradable devices have been developed and investigated as alternatives for the currently used scaffolds, drug delivery system and permanent implanted devices for optimization purpose. Because of their good biodegradability and biocompatibility, aliphatic biodegradable polyesters, mainly including polyglycolic acid (PGA), polylactic acid (PLA) and their random block copolymers poly(lactide*-co*-glycolide) acid (PLGA), have been the most widely used synthetic degradable biomaterials for biodegradable devices approved by the US Food and Drug Administration [1–4] (Fig. 1).

With respect to the chemical and mechanical properties [5–11] as shown in Table 1 and their good processabilities, PGA, PLA and PLGA have been developed for different prospective commercial applications. In the latter half of 1960s [12], aliphatic biodegradable polyesters were first utilized for synthetic biodegradable sutures. Since then, these polymers have been applied to fabricate temporary prostheses [13–17], 3D porous films and scaffolds [18–45] for tissue engineering, regenerative medicine, gene therapy and bionanotechnology, controlled/sustained release drug delivery system vehicles [46–64], surgical sutures and staples [65–67] for wound closure and implantable orthopedic fixation devices [68–70]. Particularly, as cardiovascular incidents are dramatically increasing, the applications in the field of heart patches [71] and percutaneous angioplasty and stenting treatment have been drawn more and more attention. As illustrated in Table 2, these polymers can be designed for coating drug-eluting stents (DESs) and manufacturing biodegradable stents (BDSs) [58, 72–85].

**Table 1.** Chemical and mechanical properties of PGA, PLA and PLGA [5–11]

	PGA	PLLA^a^	PDLLA^a^	PLGA
Crystallinity(%)	45-55	∼37	/	/
*T*_M_ (°C)	>200	∼175	/	/
*T*_g_ (°C)	35–40	60–65	55–60	/
Modulus(GPa)	12.5	∼4.8	1.9	/
Lose strength	1–2 months	2-5.6 years *in vivo*	1–2 months	50/50: 1–2 months
Mass loss	6–12 months		6–12 months	75/25: 4–5 months
				85:15: 5–6 months

*T*_M_, melting point; *T*_g_, glass transition temperature.

^a^Although PLA exists in four stereoisomeric forms: poly(L-lactic acid) (PLLA), poly(D-lactic acid)(PDLA), poly(D,L-lactic acid) (PDLLA) and meso-poly(lactic acid), only PLLA and PDLLA have been extensively used for biomedical applications so far.

**Table 2.** Application of aliphatic biodegradable polyesters in DESs and BDSs [58, 72–85]

Stent name	Manufacturer	Stent platform	Polymer
Axxess	Devax Inc.	Nickel- titaniumNitinol	Bioabsorbable, abluminal PLA
Custom NX	Xtent	Cobalt-chromium	Bioabsorbable, PLA
Supralimus (Infinium stent)	Sahajan and Medical	Stainless steel	Bioabsorbable, containing poly-L- lactide,polyvinyl pyrrolidone, polylactide-co-caprolactone, and PLGA
Excel stent	JW Medical System	Stainless steel	Bioabsorbable, PLa
NEVO	Johnson & Johnson	Cobalt-chromium	Bioabsorbable, polylactide-co-glycolide
BioMime	Meril Life Science	Cobalt-chromium	PLLA + PLGA
BioMatrix	Biosensors	Stainless steel	Abluminal PLa
NOBORI	Terumo	Stainless steel	Abluminal PLA
Orsiro	Biotronik	Cobalt-chromium	PLLA + silicon carbide layer
DESyne BD	Elixir Medical)	Cobalt-chromium	PLA
AXXESS	Devax Inc.	Nitinol	Abluminal PLA
Elixir Myolimus	Elixir Medical	Cobalt-chromium	Abluminal PLA
JACTAX HD	Boston Scientific	Stainless steel	Biodegradable abluminal PLA polymer
CORACTO	ALVIMEDICA	Stainless steel	Polylactic-co-glycolic acid copolymer
DREAMS I& II	Biotronik	Mg	PLGA
Igaki-TamaiStent	Kyoto Medical Planning Co, Ltd	No	PLLA
AbsorbBVS 1.0& 1.1	Abbott Vascular	No	PLLA
DESolve 1stgeneration DESolve2ndgeneration	Elixir Medical Corp.	No	PLLA
Amaranth	Amaranth Medical Inc.	No	PLLA
ART18ZBRS	Arterial Remodeling Tech.,	No	PLLA,PDLA
XinsorbBRS	Shandong Hua An Biotech., Co. Ltd.,	No	Poly-lactic acid, poly-∈-caprolactone,poly-glycolicacid
AcuteBRS	Orbus Neich	No	PLLA,L-latic-co-∈-caprolactone,PDLA

A better understanding of the mechanism of biodegradation and factors affecting the degradation process is critical for the design and preparation of aliphatic biodegradable polyesters and optimization of biodegradable devices. As a biodegradable device, aliphatic biodegradable polyester is supposed to maintain suitable degradation rate, appropriate integrity and mechanical properties during the degradation process to match the rates of bone healing, drug release and tissue regeneration. However, during the maintenance, the degradation rates of aliphatic biodegradable polyesters are closely related to the complex physiological and biomechanical environment from internal and external. Extensive investigations have been launched during last twenty years in view of how physiological and biochemical environment *in vitro* and *in vivo* significantly affects biodegradation process [86–95]. The mechanical load is one of the most important factors that may cause the polymer degrade not as predetermined and lead to the devise fracture. It has drawn considerable attention recently when scientists are designing, preparing and optimizing implantable orthopedic fixation devises and cardiovascular BDSs. The uncontrollable degradation rate affected by unpredicted mechanical loads may cause the orthopedic fixation plates/screws and cardiovascular BDSs degrade too fast to keep the integrity and mechanical properties to match with the bone self-healing and vessel remodeling process, making the plates/screws or stents fracture before an expected life, which may result in bone refracture, blood vessel elastic recoil or distal vascular blockage by stent fragments. Hence, a lot of studies on effects of different mechanical loads on the degradation of aliphatic biodegradable polyesters have been carried out yet, but no systematic summary has been done.

The objective of this article is to outline the mechanism of biodegradation and several different mechanical loads that have been reported to affect the biodegradation process. Other physiological and biochemical factors related to mechanical loads will be also discussed.

## Mechanism of biodegradation

**Figure 2.**
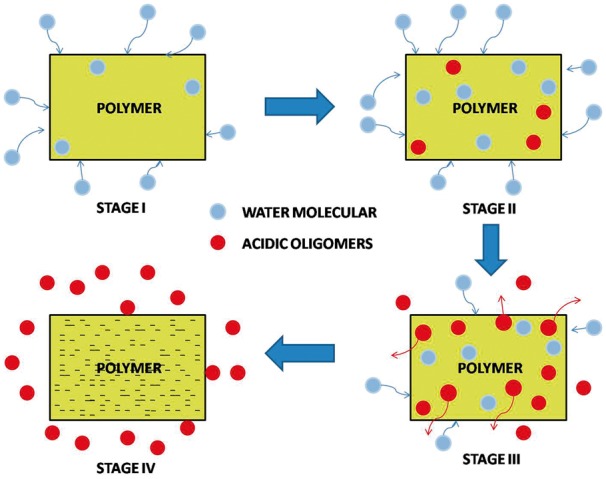
Schematic representation of hydrolytic degradation of polymer

It has been evidenced that there are hydrolytically labile chemical bonds in the backbone of PGA, PLA and PLGA, so these polymer primarily undergo bulk degradation *in vivo* via the chemical random scission of the hydrolytically unstable ester backbone into lactic acid or glycolic acid (GA) monomers, which can be broken down into carbon dioxide and water in the urine and eliminated from the body safely by the tricarboxylic acid cycle [96]. As shown in Fig. 2, the biodegradation process is elucidated to complete in four consecutive steps [97–100]: (i) Hydration. The aqueous medium penetrates the polymer matrix and disrupts the secondary forces, which lead to the relaxation and the decrease of the glass transition temperature [101]; (ii) Initial degradation. After hydrolysis, in the hydrated region of the polymer, the cleavage of the covalent bonds in the polymer backbone begins, resulting in the molecular weight decrease of the polymer. As hydrolysis goes on, the hydrolysis reaction inside the polymer matrix were auto-catalyzed by more and more carboxylic end-groups [102], leading to the continuously decrease of the molecular weight of the polymer. The polymer loses its mechanical strength along with the decrease of the molecular weight, but the integrity of the polymer maintains. (iii) Further degradation. The molecular weight of the polymer keeps falling to a threshold that the integrity of the polymer no longer can be held [97]. So, significant mass loss begins. (iv) Solubilization or erosion [103]. The polymer loses its weight and the fragments are further cleaved to molecular which are soluble in the medium [97].

## Effects of mechanical loads

After implantation, the degradation rates of biodegradable medical devices such as orthopedic fixation devices, cardiovascular stents, grafts and heart valves which are composed of aliphatic biodegradable polyesters have been reported to be affected by various local and gross mechanical loads from different surrounding tissues, with conflicting results. On the contrary, the micro and macro structural, mechanical and morphological properties of aliphatic biodegradable polyesters have also been influenced during the degradation process.

### Tensile, compressive and cyclic loads

**Figure 3.**
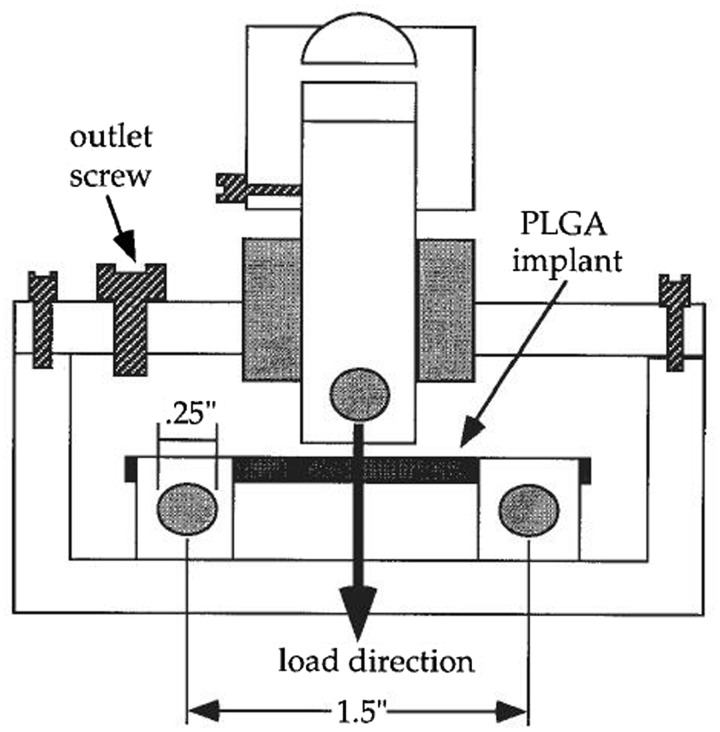
Schematic diagram of a chamber used to load a PLGA implant in three-point bending. The implant rests on two roller end supports and is loaded at its center, vertically downward by a plunger. The magnitude of the plunger displacement can be varied. (Reproduced from ref. [111], with permission from Wiley)

**Figure 4.**
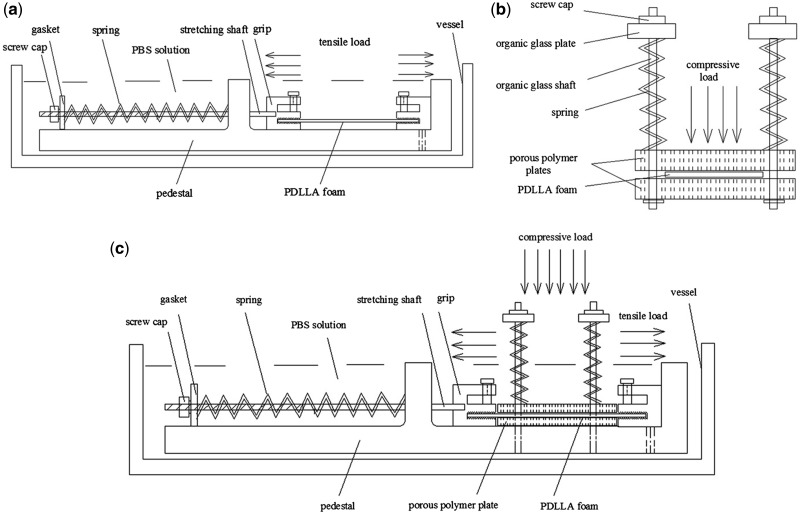
Self-made load-providing devices: **(a)** tensile load-providing device; **(b)** compressive load-providing device and **(c)** tensile-compressive combined load providing device. (Reproduced from ref. [86], with permission from Elsevier)

**Figure 5.**
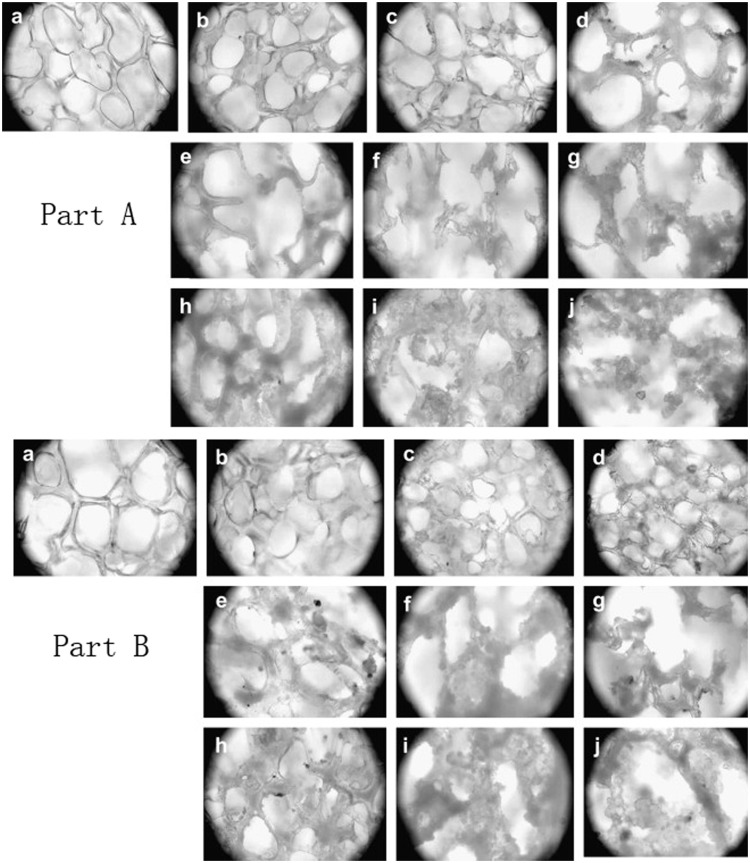
Morphologies of PDLLA before and after degradation (magnification of 200×) Part **(A)**: tensile loaded (15 N) and compressive loaded (100 N): (a) before degradation; unloaded degradation after (b) 1 month, (c) 2 months and (d) 3 months; tensile loaded degradation after (e) 1 month, (f) 2 months and (g) 3 months; tensile-compressive combined loaded degradation after (h) 1 month, (i) 2 months and (j) 3 months. Part **(B)**: tensile loaded (25 N) and compressive loaded (100 N):(a) before degradation; unloaded degradation after (b) 1 month, (c) 2 months and (d) 3 months; tensile loaded degradation after (e) 1 month, (f) 2 months and (g) 3 months; tensile-compressive combined loaded degradation after (h) 1 month, (i) 2 months and (j) 3 months. (Reproduced from ref. [86], with permission from Elsevier)

The effects of tensile, compressive and cyclic loads, as the most common types of mechanical loading *in vivo*, on the degradation process have been extensively investigated. Bikales [104] first proposed that mechanical stresses may accelerate the chain scission, crosslinking and other changes in biodegradable polymers’ chemical and physical properties. Otherwise, these changes may influence the mechanical properties of polymers substantially. Miller and William [105] demonstrated that the degradation rate of PGA sutures was dependent on the magnitude of a pre-imposed strain. As reported, the degradation of PGA sutures characterized by the changes in the tensile load at break was considerably enhanced both *in vivo* and *in vitro* by pre-straining the specimen to one half of the normal extension at break. Daniels [106] reported that the cyclical mechanical stress could accelerate the degradation rates of several polymers. Then a test methodology was developed for poly(ortho ester) to characterize the effect of a simulated mechanical and chemical body environment with aerated tris-buffered saline (pH 7.4 and 37°C) on the degradation rate, mainly focusing on the changes of the stress-strain behavior. The results showed that cyclic loading in air alone had no effect on the rate of the change of the mechanical property. However, the flexural yield strength decreased by 29% in static load group and 75% in cyclic loading group respectively, while the modulus of elasticity reduced to 80% and 25% of the original value in static load group and cyclic loading group separately after 40 days when specimens exposed to tris-buffered saline simultaneously. This is the first attempt to investigate multiple factors including pH, oxygen concentration, temperature and mechanical loads [107]. However, in contrary, the cyclic tensile loading presented no effect on the degradation of a PLA–PGA copolymer in Agrawal and Kennedy’s work [108]. Zhong *et al.* [109] found that 4% applied strain increased the degradation rate of a PLA/PGA copolymer compared with unloaded samples both in the water and hydrogen peroxide solution. Thompson *et al.* [110] examined the *in vitro* mechanical properties of a PLA/PGA (50/50) two phase implant under a cyclic compressive load over 6 weeks compared with no loading conditions. The dynamic compressive load collapsed the pores in the polymer matrix, resulting in a reduction in volume, so the more compact structure presented a smaller surface area for hydrolysis. Though the manifestation that the polymer underwent a surface deformation to be more stiffness occurred, there was a threshold that the polymer could no longer maintain the mechanical properties and started to collapse as hydrolysis broke down the polymer chains. A cyclic three-point bending loading of 720 cycles/day at 0.4 Hz for 2 weeks was conducted by Arm and Tencer [111] utilized a self-design chamber shown in Fig. 3 to biodegradable PLGA cylindrical implants. But there was no significant change in their mass loss nor swelling and molecular weight during the period. Remarkably, the superficial pores in the highest stress region were elongated into cracks. This demonstrated that the pores probably acted as stress risers to initiate cracks. Besides, the pore and crack density was greater for loaded implants, but no relation with the magnitude of deformation was found. Fan *et al.* [86] investigated the mechanism of how the different continuous loads affected the hydrolytic degradation of poly(D,L-lactic acid) (PDLLA) foam gasket in phosphate-buffered saline (PBS) solution (pH 7.4 at 37°C) by the self-made load-providing devices shown in Fig. 4. Two different magnitudes of tensile loads (15 N and 25 N) combined with 0 and 100 N compressive loads were used to mark the changes of the surface morphology, molecular weight, elastic modulus, tensile strength and mass loss when compared with those with no load. Within 3-month observation, it has been concluded that the mechanical load played an important role in accelerating the degradation rate. The load-induced degradation rate of polymers was faster than the rate of unloaded ones and the combinative load affected the rate more distinctly. The changes in Morphologies of PDLLA were shown in Fig. 5. Afterward, similar work about the degradation behavior of porous PLLA/*β*-TCP and PLGA/*β*-TCP composite scaffolds under the dynamic loading and static condition in PBS solution (pH 7.4 at 37°C) for 12 weeks was examined by Kang [87] and Yang [24]. The dynamic loading condition accelerated the degradation process with respect to more rapid reductions in mass, height, diameter and number-average relative molecular mass compared with that under the static conditions with no stress. Similarly, with the same methods, the cyclic loading was also found to accelerate the *in vitro* degradation of porous PLGA scaffolds incubated in PBS solution (pH 7.4 at 37°C) for 12 weeks, accounting for the faster mass loss, dimensions and shape change, morphological variations and reduction in mechanical properties [88]. After that, Li *et al.* [89] demonstrated that the tensile elastic modulus and ultimate strength of electrospun PLGA scaffolds in tensile loaded group decreased faster than that with no load, after a dramatical increase in both groups, during the 7-week degradation in PBS solution (pH 7.4 at 37°C). Moreover, changes in their molecular weight, thermal properties, lactic acid release and morphology property indicted the tensile loading accelerate the degradation rate. In addition, Zhao *et al.* [90] reported the accelerated degradation of electrospun PLLA membranes when subjected to the cyclic stretch loading in Tris-HCl buffer solution containing proteinase K. Furthermore, a quantitative investigation of the tensile stress and *in vitro* degradation rate of PLGA membranes has been conducted by Guo *et al.* [91]. Tensile stress in levels of 0.1–0.5 MPa and deionized water was applied. As the magnitude of tensile stress increased, more loss in the mass and mechanical properties, elastic modulus and tensile strength, were observed.

### Fluid shear stress

**Figure 6.**
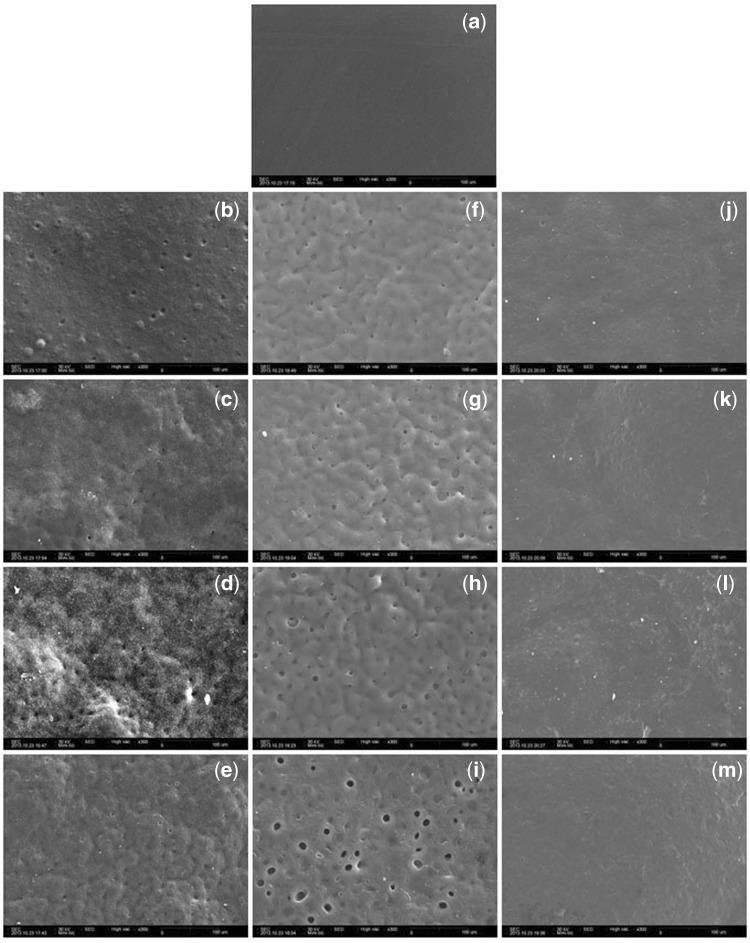
PLGA morphology before and after degradation with different fluid shear stress (magnification of 300×). **(a)** before degradation. **(b–e)** unloaded degradation after (b) 5 days, (c) 10 days, (d) 15 days and (e) 20 days. **(f–i)** at a fluid shear stress of 12 dyn/cm2 after (f) 5 days, (g) 10 days, (h) 15 days and (i) 20 days. **(j–m)** at a fluid shear stress of 30 dyn/cm^2^ after (j) 5 days, (k) 10 days, (l) 15 days and (m) 20 days. (Reproduced from ref. [94], with permission from Wiley)

Fluid shear stress is one type of the main mechanical loadings generated by fluid flow and also has been proved to be effective to the degradation rate. Agrawal *et al.* [92] examined the effects of fluid flow of 0.25 ml/min on the *in vitro* degradation characteristics and kinetics of PLA-PGA scaffolds with different porosity and permeability in PBS solution (pH 7.4 at 37°C) for up to 6 weeks. The changes in mass, molecular weight and elastic modulus indicated that the increasement of porosity/permeability and fluid flow could decrease the degradation rate significantly. This can be attributed to the mass transportation of fluid flow and the autocatalysis of the degradation reaction generated by the acidic degradation products, although the fluid shear stress is too small and negligible. Besides, a much clearer comparative study was done by Huang *et al.* [93] on the degradation of PLGA 50/50 cylinder subjected to Hank’s simulated body fluid (Hank’s SBF) under static and body fluid flow condition. Significant decrease of weight-average molecular weight began rapidly in static SBF but this happened until 10 days in the dynamic system. Moreover, significant mass loss occurred from 20 days in the static condition while little changed in the dynamic one during the 30 days. With respect to the morphology change, a slower degradation rate in the dynamic system was indicated. Furthermore, Chu *et al.* [94] did a series of quantitative work on the effect of different steady fluid shear stresses on the degradation of PLGA in deionized water (pH 7.4 at 37°C) for 20 days. The viscosity of the degradation solution in the loaded condition subjected to fluid shear stress was more severely affected. Raising the fluid shear stress could speed up the loss of ultimate strength and slowed down the decrease of tensile elastic modulus as well. Similarly, the fluid shear stress did have effect on the morphology change as shown in Fig. 5. Subsequently, the effect of different patterns of fluid shear stress on the degradation was investigated [95]. Steady, sinusoid and squarewave fluid shear stress with the same average magnitude and the different maximum fluid shear stress and ‘window’ of effectiveness were applied. The results showed that the maximum fluid shear stress accelerated the loss of molecular fragments in the solution while the ‘window’ of effectiveness affected as well in the early stage. In addition, the maximum fluid shear stress and ‘window’ of effectiveness accelerated the reduction of tensile modulus and ultimate strength while the maximum fluid shear stress acted the leading role in the decrease of tensile modulus at the early degradation stage. However, there was no clear evidence showing that different patterns of fluid shear stress influenced the morphology property (Fig. 6).

## Factors related to mechanical loads

It’s worth noting that only the factor of mechanical loads in all researches aforementioned was considered due to single factor analysis method. But it is well known that the degradation rates are difficult to be ideal because of the inherent properties and complex environmental factors *in vivo*. The degradation process suffers a combined impact of mechanical loads and these factors. So understand the effect of each variable on the degradation rate is the foundation to evaluate the degradation process *in vivo* under the condition of multiple factors.

### Inherent physical factors

Accordingly, several inherent properties are important factors that affecting the degradation rate, including the copolymer composition, molecular weight, shape, and indirect factors of glass transition temperature and crystallinity which are dependent on the copolymer composition.

#### Copolymer ratio

Miller *et al.* [112–113] first examined the rate modification with the changes in copolymer ratios and confirmed that PLGA 50/50 was very hydrolytically unstable. After that, Park [114] prepared a wide range of PLGA microspheres with different copolymer compositions with no active ingredients. The degradation behaviors of PDLLGA 90/10, PDLLGA 80/20, PDLLGA70/30, PDLLGA50/50 and PDLA were compared in an Eppendorf centrifuge tube incubated at 37°C with PBS up to 53 days. As reported, the hydrolytic scission preferentially occurs between the ester bonds linked with the GA unit (glycolic–glycolic acid or glycolic–lactic acid).Similarly, Wang and Wu [115] studied the degradation process of three different PLGA samples with the ratio of 46/54, 65/35 and 72/28. The results showed a positive correlation between the mass loss and increase of GA residue in the oligomers. Afterwards, they [116] reported a systematic study of the effect of copolymer composition. With similar molecular weights, PLGA 50/50, 65/35, 75/25, 85/15 and PLLA were compared. The absolute value of the biodegradation rate constants were evidenced to rise with increasing the GA content. This is in clear agreement with the results reported by Li [117]. In summary, due to the great hydrophilicity, the ester bonds linked with GA unit affect the degradation rate and there is a positive correlation between the content and the rate.

#### Molecular weight

Park [114] also examined the degradation behaviors of two PDLA microspheres with molecular weight of 17 and 41 kDa respectively. The results exhibited that the degradation behaviors were greatly depended on the molecular weight of raw PDLA during the 53-day incubation. Microspheres with the lower molecular weight showed a significant degradation with reduced *T*_g_. However, because of their glassy state, microspheres with the higher molecular weight show no detectable change during in the 53 days’ degradation. Wang *et al.* [118] investigated the effect of molecular weights of 1317 and 3025 Da on the biodegradation of two different LGA oligomers 72/28 in tubes incubated at 37°C with PBS (pH 7.4) shaking at 30 rpm. A slower weight loss of LGA oligomer with the higher molecular weight was found than that having the lower molecular weight counterpart. On the contrary, Cam *et al.* [119] used four PLLAs with different molecular weights of 300, 450, 650 and 3000 kDa to study the effect of molecular weight on degradation in 0.01 _N_NaOH alkaline solution (pH 11.8) at 37°C. The crystallinity of samples decreased from 30 to 3% with an increase in molecular weight. The films had higher molecular weight prior to hydrolysis and degraded at a higher rate. Another study done by Wu and Wang [116] investigated a group of PLGAs with the same composition of 75/25 but different molecular weights of 12 876, 31 403, 66 946, 124 450 and 166 630 Da, respectively. The first order biodegradation reaction rate constant observed were 0.0472, 0.0681, 0.0834, 0.0961 and 0.0969 day^−^^1^separately. After the initial stage, PLGA with higher molecular weights degraded faster than those with lower ones. All above, the molecular weight has a considerable effect on the biodegradation rate in three ways. First, lower molecular weight polymers have more carboxylic end groups per unit weight and are more hydrophilic than higher molecular weight counterpart. Second, the *T*_g_ is frequently influenced by molecular weight. Higher molecular weight polymers usually have higher *T*_g_ than 37°C [120]. Third, the higher molecular weight polymers have longer polymer chains. The chances being attacked by water molecules is increased because of the longer chains [121].

#### Shape

Li *et al.* [122–126] investigated the degradation of PLA and PDLLA parallelepiped devises and found, for the first time, that the degradation process was significantly faster in the inner part than at the surface both *in vivo* and *in vitro* [127]. Grizzi *et al.* [128] reported that instruments with dimensions smaller than the thickness of the more stable outer layer could degrade slower than larger ones and they testified this hypothesis on compression moulded plates, millimetric beads and submillimetric microspheres and cast films. A critical thickness of 200–300 μm was proposed. Similarly, Witt and Kissel [129] compared the degradation rates of microspheres, films, rods and tablets with different dimensions but the same material of PLGA 50/50, and the apparent constant rate of degradation were shown to be 0.041, 0.093, 0.115 and 0.1035 day^−^^1^, respectively. Lu *et al.* [130] also reported that thick films degraded faster than thin ones and indicated that the degradation rate of porous foams could be designed by differing the pore wall thickness and pore surface/volume ratio [131] for the use of tissue engineering scaffolds. He and Xiong [27] investigated the *in vitro* degradation process of three-dimensional porous and films made from PLGA 85/15 and demonstrated that the films degraded much faster. It can be reasonably concluded that, due to acid catalysis of carboxylic end groups, the degradation rate of aliphatic biodegradable polyesters can be affected by shape.

### Environmental factors

Some biochemical environmental factors such as pH value and temperature were evidenced to affect the rate as well. Belbella *et al.* [132] proved that degradation of PDLLA was related to the pH value (pH value of 2.2, 4.2, 6.0, 7.4, 8.4 and 10.1 were used) and the hydrolysis was much more catalysed at acidic and alkaline pH than at neutral one. Wang *et al.* [118] found that the degradation of the LGA oligomer 72/28 is faster in phosphate buffer (pH 7.4, 0.2 M) than in Na_2_B_2_O_7_ 10 H_2_O buffered solution (pH 9.4, 0.1 M). Holy *et al.* [133] demonstrated that the rate of macroporous PLGA 75/25 was much faster in pH 5.0 than in pH 6.4 and 7.4 after 16 weeks of *in vitro* degradation. Wu and Wang [116] also examined the degradation of PLGA 50/50 with a weight-average molecular weight of 13134 D in three different buffers including pH 5.0 phosphate buffer (0.2 M), pH 7.4 phosphate buffer (0.2 M) and pH 9.24 sodium borate buffer (0.1 M). The results showed that the biodegradation rate decreased when the pH was 9.24 while increased in an acidic one (pH 5.0) from the third week. This is in agreement with the result reported by Yoo [134].This can be concluded that aliphatic biodegradable polyesters degrade faster in acidic medium than in alkaline or neutral one. 37 and 100°C were applied by Jamishidi [135] to study the effect of temperature on the degradation behavior of PLLA fibers in PBS. The tensile strength was observed reducing to half at 100°C after 10 h while no changes was observed at 37°C. In agreement, Aso *et al.* [136] reported that the molecular weight of PDLLA discs and microspheres decreased rapidly at 50°C. In Belbella’s work [132], the degradation of PDLLA nanospheres at pH 7.4 was much faster at 37°C than at 4 and −18°C. In addition, Hakkarainen *et al.* [137] also reported a dramatic acceleration of degradation of PLLA and PLGAs at 60°C. As such, the degradation rate of aliphatic biodegradable polyesters is highly dependent on the temperature, especially when it is higher than the glass transition temperature of polymers. Deng [138, 139] also found that an elevated temperature would accelerate the degradation process of 90/10 poly(glycolide*-co*-L-lactide) multifilament braids in PBS solution.

Besides, other environment factors including the addition of drug [140–143], sterilization [144–147] and enzymes [148–157] and so on are reviewed by Alexis [121] and a lot of these facts presented controversial results in so far.

## Conclusion and prospects

In general, though the mechanical load may not be able to initiate the degradation process independently, it is reasonable to conclude that the mechanical load can influence the degradation of aliphatic biodegradable polyesters. The mechanical load can get the polymer extended for more cavities. Therefore the water molecular can be much easier to diffuse into the inner part to scissor the chain segments, leading to a faster hydrolysis. Then, under the action of stretch or compression, the conformational strain energy change might change the length or angle of the bonds, resulting in weakening of the stability. Furthermore, the load could affect the intrinsic mechanical properties of the polymer. Besides, the fluid shear stress of different patterns with the maximum fluid shear stress and the ‘window’ of effectiveness could accelerate the loss of ultimate strength and delay the decrease of tensile elastic modulus. The conclusions all above indicated that investigations into the effects of mechanical loads on the degradation should be very indispensable for appropriately designing and preparing not only aliphatic biodegradable polyesters but also other biodegradable polymers for targeted applications.

Till date various studies about one of the various physiological and biochemical factors have been carried out. However, the degradation rates of aliphatic biodegradable polyesters suffer a combined impact of mechanical loads and other complex inherent and environmental factors *in vivo*. It can be anticipated that more *in vivo* experiments on the degradation behavior under a single kind of mechanical loads and more combination condition of mechanical loads and multiple factors should be considered during the elucidating process of the degradation behavior in future *in vitro* work.

It is much urgent to propose the mechanism of degradation of aliphatic biodegradable polyesters affected by combined factors both *in vitro* and *in vivo*, which is the foundation to keep the degradation rate controllable and evaluate the degradation process *in vivo* accurately. Only then can the degradable devise achieve the desired effects and further expand the special applications of aliphatic biodegradable polyesters.

## Funding

This work was supported by the National Key Technology R&D Program (Nos. 2014BAI11B02, 2014BAI11B03, 2012BAI18B01), National Natural Science Foundation of China (Nos. 11120101001, 11421202, 31370959, 11572029, 31470915), National key research and development program in China (No. 2016YFC1100704, 2016YFC1102202, 2016YFC1101100), Beijing Nova Programme Interdisciplinary Cooperation Project (No. xxjc201616), Key Laboratory of Advanced Materials of Ministry of Education of China (Tsinghua University), Fok Ying Tung Education Foundation (No. 141039) and International Joint Research Center of Aerospace Biotechnology and Medical Engineering, Ministry of Science and Technology of China, and the 111 Project (No. B13003).

*Conflict of interest statement*. None declared.
